# Staphylococcal Enterotoxin A Induces Small Clusters of HLA-DR1 on B Cells

**DOI:** 10.1371/journal.pone.0006188

**Published:** 2009-07-09

**Authors:** Kedar Narayan, Edward M. Perkins, Gavin E. Murphy, Sarat K. Dalai, Michael Edidin, Sriram Subramaniam, Scheherazade Sadegh-Nasseri

**Affiliations:** 1 Graduate Program in Immunology, Johns Hopkins Medical Institutions, Baltimore, Maryland, United States of America; 2 Department of Biology and Integrated Imaging Center, Johns Hopkins University, Baltimore, Maryland, United States of America; 3 Laboratory of Cell Biology, Center for Cancer Research, National Cancer Institute, Bethesda, Maryland, United States of America; 4 Department of Pathology, Johns Hopkins Medical Institutions, Baltimore, Maryland, United States of America; New York University School of Medicine, United States of America

## Abstract

The superantigen SEA causes non-specific hyperactivation of T and B cells at low concentrations. Studies of mutants or soluble proteins suggest SEA is bivalent for its ligand, MHC class II. However, the interaction between these molecules on intact cells is unknown. On primary mouse B cells expressing the MHC class II allele HLA-DR1, measurements of Förster Resonance Energy Transfer between HLA-DR1 molecules on SEA-treated cells indicated specific clustering, not observed in untreated or monovalent superantigen treated cells. Tomographic visualization and electron microscopy of immunogold-labeled SEA-treated B cells revealed small clusters of surface HLA-DR1 (≤4 gold labels). These results present direct visual evidence of SEA-mediated clustering of MHC class II molecules on treated antigen presenting cells, and provide a new structural approach to addressing problems of this nature.

## Introduction

The term “Superantigen” is used to define endogenous or exogenous factors that can stimulate T cells whose T Cell Receptors (TCR) bear specific Vβ domains, irrespective of the composition of the rest of the receptor [1], resulting in the stimulation of a large fraction (up to 20%) of the T cell population [2]. Some of the most potent exogenous superantigens (SAgs) known to man are the enterotoxins secreted by Staphylococcal bacteria. Staphylococcal Enterotoxins (SEs) are a family of structurally related basic secretory proteins that are important virulence factors for the pathogen. By mediating massive cellular proliferation and cytokine secretion at extremely low concentrations (5,6), these SAgs can cause systemic pathology in the host, ranging from nausea and fever up to toxic shock and death [3], [4]. SEs can bind relatively non-polymorphic regions outside the peptide binding groove of MHC class II molecules on Antigen Presenting Cells (APCs) [5], [6], [7] as well as conserved Vβ regions of TCR molecules [8], [9], [10], leading various groups to hypothesize that these SAgs may act as a binding “bridge” between MHC class II and TCR, resulting in downstream signaling events and immune activation [11], [12], [13].

One of the most potent SEs is Staphylococcal Enterotoxin A (SEA), with an exceedingly low half-maximum stimulating dose of 0.1 pg/mL [2]. SEA is somewhat atypical in that it has two binding sites for two corresponding sites on MHC class II, a high affinity Zinc coordinating site on the β chain of MHC class II [14] and a second weaker (>1 µM affinity) binding site on the α chain that has been shown to play an important role in the complete functional activity of SEA [15], [16]. Studies have suggested a cooperative model where the binding of one SEA to MHC class II favors the binding of the second SEA molecule [13], [17], [18], and MHC class II - (SEA)_2_ trimers have been isolated in solution [18]. These results have led to the speculation that SEA could crosslink multiple MHC class II molecules on the surface of APCs. Indeed, when MHC class II expressing cell lines were treated with SEA, but not with mutants missing either binding site or toxins with one MHC class II binding site, downstream signaling [19], [20], inflammatory cytokine gene upregulation [19] and homotypic aggregation [21] was observed, even in the absence of T cells. These results hint a role for a multivalent binding mode between SEA and MHC class II, and indeed, many subsequent studies on superantigens have assumed this multivalency of SEA as part of its functionality. However, the actual membrane reorganization of MHC class II on the surface of a cell in response to SEA treatment has not been directly probed, and as such, remains unknown.

In this study, using advanced optical spectroscopic and electron microscopic techniques we demonstrate that the binding of SEA via both of its binding sites to MHC class II on the surface of primary B cells induces small clusters of these molecules. Working with transgenic mice developed in our lab that express only the MHC class II allele HLA-DR1 (DR1), we measure Förster Resonance Energy Transfer (FRET) between antibody-labeled DR1 molecules. We show that the addition of SEA results in the increased clustering of DR1 in a dose dependent manner, and is dependent on both binding sites. We employ electron tomography, which allows us to directly visualize the three-dimensional distribution of immuno-gold-labeled DR1 molecules on the surface of these B cells at high resolution, as well as immuno-gold labeling followed by 2D transmission electron microscopy. We observe a significant increase in small-scale clustering of MHC class II on SEA treatment over controls. These results provide evidence that SEA likely mediates the formation of small MHC class II clusters or “daisy-chain oligomers” on the B cell surface, and does not aggregate or coalesce large numbers of MHC class II.

## Methods

### SDS-PAGE

SDS-PAGE experiments were performed essentially as previously described [22]. 1 µM wtDR1 was incubated in the absence or presence of 50 µM of the immunodominant peptide derived from Influenza Hemagglutinin HA_306-318_ (PKYVKQNTLKLAT) or its synthetic variant HA_Y308A_ (PKAVKQNTLKLAT) at 37°C for 48 h in PBS+10 µM ZnCl_2_ pH 7.4. The wtDR1/HA_Y308A_ complexes were incubated for an additional hour with a molar equivalent of soluble SEA or SEH before mixing with equal volumes of SDS-PAGE sample buffer containing 0.2% SDS and incubating for 10 min at room temperature. These samples were then applied to 12% PAGE gels, and the gels were silver stained according to standard protocols.

### Surface Plasmon Resonance measurements

Cys-HA_306–318_ peptides were immobilized on an SMPB activated CM5 chip in a BIAcore 2000 instrument and wtDR1/HA_Y308A_ complexes (∼2.5 µM) were allowed to bind to the peptide surface for ten minutes [23]. Complexes were allowed to dissociate for approximately three minutes, after which a solution of wtDR1/HA_Y308A_ complexes (∼2.5 µM) pre-incubated with SEH or SEA at a 1∶1 molar stoichiometry was injected over the surface of flow cell 1 or 2 respectively for another ten minutes. To control for DR1 binding to freshly dissociated peptides on the chip, a second injection of wtDR1/HA_Y308A_ alone was performed on a control flow cell. In all cases a quick spin in a G-50 column equilibrated with PBS+10 µM ZnCl_2_ was performed to remove excess peptide and to exchange buffer. RUs were measured ∼3 seconds after end of injections to exclude the common artifact of sudden RU changes caused by small changes in pH or protein or ion concentrations. The linear association curves were caused by mass-transport binding as a result of low analyte concentration in the injection solution. The running buffer was PBS+10 µM ZnCl_2_, pH 7.4, and flow speed was maintained at 5 mL/min.

### Labeling of Antibodies

The anti-HLA-DR probe used in all experiments was the mouse IgG1 monoclonal antibody L243. The antibodies were labeled with Cy3 or Cy5 fluorophores (Amersham Biosciences, UK) according to the manufacturers' protocols. The labeled antibodies were separated from excess dye using a size-separation column and stored at 4°C. The Dye-to-protein (D/P) ratios were approximately 2.5∶1 for all experiments. There were no noticeable aggregates in the size exclusion profile, and the labeled antibodies were used for experiments within three months of preparation.

### Affinity measurements of antibodies

Single cell suspensions of fresh DR1 transgenic mouse splenocytes (see below) were incubated with 2.5 µg soluble SEA, SEH (Toxin Technologies, CA) or no enterotoxin per 1×10^6^ cells for 30 minutes, washed and stained with doubling dilutions of Cy3 conjugated L243. After washing, the cells were then analyzed by flow cytometry on a FACSCalibur (BD Biosciences). The Mean Fluorescence Index (MFI) of gated cells was then plotted against Antibody concentration, and the data fit to a simple saturating binding curve, as described by the equation
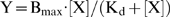
where Y was the MFI value measured by flow cytometry, B_max_ is the maximal binding at equilibrium, X is the antibody concentration, and K_d_ is the equilibrium dissociation constant.

### Mice and cells

In all experiments, male wtDR1 transgenic mice (C57BL/6 background) of age 8–10 weeks were used as the source of cells. These mice are normal and express the human class II allele HLA-DR1 as their only MHC class II molecule at normal levels of expression (Dalai SK and Sadegh-Nasseri, unpublished data). Single cell suspensions were generated from naïve spleens, and B cells (>90% purity, **Supplementary Fig. S2**) were generated by negative selection using CD43 magnetic beads (MACS, Miltenyi Biotec) according to the manufacturers protocols. After washing and resuspending in media, the cells (usually 1×10^6^ purified B cells per group) were incubated with various concentrations of the specified enterotoxin in media for 45 minutes at 37°C, washed in FACS buffer (PBS pH 7.4+1% FCS+0.005% NaN_3_), and then prepared for FRET or EM measurements, as detailed below. All mice were housed in a Johns Hopkins University animal facility under virus-free conditions. All experiments involving mice were performed with protocols approved by the Animal Care and Use Center of the Johns Hopkins School of Medicine.

### Confocal Microscopy and FRET measurements

#### Preparation of cells

The following steps were performed at 4°C and in FACS buffer unless otherwise specified. Freshly isolated B cells that were previously incubated either with or without enterotoxin at 37°C were incubated with anti-Fc antibodies for 20 minutes, washed, and incubated for 1 hour with saturating concentrations of fluorophore conjugated L243. Ratios of 1∶1, 1∶2, or 1∶4 Donor (Cy3): Acceptor (Cy5) were used. Care was taken to keep total antibody concentrations constant and well above saturation (∼2 µM). The cells were then washed and resuspended in PBS, then fixed in 4% fresh paraformaldehyde at room temperature for 30 minutes. To test for capping by confocal microscopy or electron microscopy, negatively selected B cells were incubated with saturating concentrations of either unconjugated L243 or L243-FITC, washed, and incubated with Gold-labeled or unlabeled goat anti-mouse IgG polyclonal antibodies at 37°C, respectively. To test for internalization, B cells were incubated with SEA for various lengths of time at 37°C and stained with L243-FITC at 4°C.

#### Microscopy

All the experiments for this section were performed on a Zeiss LSM 510 Meta confocal microscope at the Integrated Imaging Center, Johns Hopkins University, and the data analyzed on Zeiss Analysis software. The fixed B cells were allowed to rest on cover slips and then imaged at 63× magnification using a Zeiss Apochromat objective with averaging factor of 4 to increase the Signal-to-noise ratio. Emission from Cy3 and Cy5 were detected using appropriate filter sets (Cy3: excitation 543 nm and emission Band Pass 560–615 nm. Cy5: excitation 633 nm and emission Band Pass 646–753 nm of the META spectral detector). A multi-time bleach macro was utilized to run the following loop: *(i) pre-bleach* - images of several Cy3 and Cy5 labeled B cells were recorded in their respective channels; *(ii) photobleach* - the acceptor Cy5 on these cells was photobleached using the 633 nm laser within a region of interest (ROI). The photobleach program was set up to achieve >85% bleaching of the acceptor (the donor was not bleached); *(iii) post-bleach* - the same cells were imaged again using the same parameters as the pre-bleach, and the fluorescent intensities in all cases quantified.

Several iterations of the loop were executed to image as many cells as possible at various (x,y) coordinates on the cover slip. In most experiments at least 50 cells for each Donor: Acceptor (D∶A) ratio per group were imaged and analyzed. The quantified intensities of various ROIs in the donor and acceptor channels were then used for the calculation of FRET efficiencies using software provided by Zeiss. Cells that were only Donor labeled, irrespective of experimental group, showed no FRET as measured by Acceptor Photobleach experiments (data not shown).

### Immunolabeling and Transmission Electron Microscopy

#### Preparation of cells

Fresh B cells were prepared and incubated with enterotoxin as above; here, after blocking with anti-Fc antibodies the cells were incubated with saturating concentrations (∼5 µM) of unlabelled L243 for 1 hour at 4°C. In the case of the EM experiments, either 5 or 10×10^6^ purified B cells were used per group. After washing, the cells were fixed in 4% paraformaldehyde, washed twice in PBS+2.5% FCS and incubated overnight at 4°C in saturating concentrations of donkey anti-mouse antibodies conjugated to 6 nm Gold Beads (Jackson ImmunoResearch Labs, PA) and washed again.

#### Microscopy

The cells were fixed in 3% Paraformaldehyde+1.5% Glutaraldehyde, post-fixed in OsO_4_, pelleted, embedded and prepared as previously described [24]. The block containing the cell pellet was cut to fit in a Leica UCT ultramicrotome and thin sections (80 nm) were collected on uncoated grids, and post-stained with lead citrate and uranyl acetate. Projection electron microscopic images were recorded at magnifications in the range between 45000× and 54000× using a Philips EM 410 or 420 transmission electron microscope equipped with a SIS Megaview III CCD digital camera and analyzed using the AnalySIS software (Olympus).

#### Distance Measurements

Using “blind” samples, at least 30 slices per group were examined. The distances between adjacent gold beads were then measured along the contour of the cell membrane (this is not possible for the 3D images, as the gold beads are rendered as points in space). It is important to note that this measure of clustering is unable to quantitatively determine the number of MHC class II molecules clustered primarily because technical limitations with antibody staining preclude a labeling efficiency of 100%. Also, the 2-D measurements do not account for the width of the sections. However, care was taken to keep the sections thin (80 nm) and of the same thickness across the groups. In addition, all groups were treated and measured in exactly the same way, and the experiments were conducted blind. Hence, this is a valid method to compare relative differences in cell surface distribution of DR1 between those groups, within an experiment.

### Electron Tomography

250 nm thick sections were prepared in the same way as described above for the thin sections. 15 nm gold fiducial beads were deposited on the grids to aid alignment of tilt series. Collection of tilt series and reconstruction of tomograms was performed as previously described [25]. Briefly, a series of projections of the region of interest were taken at various tilt angles (−60° to +60°, 2° increments) and recorded using a 2k×2k CCD (**Supplementary Movie S1)**. The resulting images were back-projected and aligned using the IMOD package (IMOD, etomo [26], [27]) to generate the tomogram (**Supplementary Movie S2**). The tomograms were segmented using Amira (Amira 3.1, Mercury Computer Systems GmbH, Berlin, Germany); the cell membrane and the electron dense gold beads are highlighted in purple and as golden spheres, respectively, to generate the final images and/or movies (**Supplementary Movie S3**).

## Results

### SEA and SEH bind DR1 and confer SDS stability to peptide-DR1 complexes

**Figure 1:**
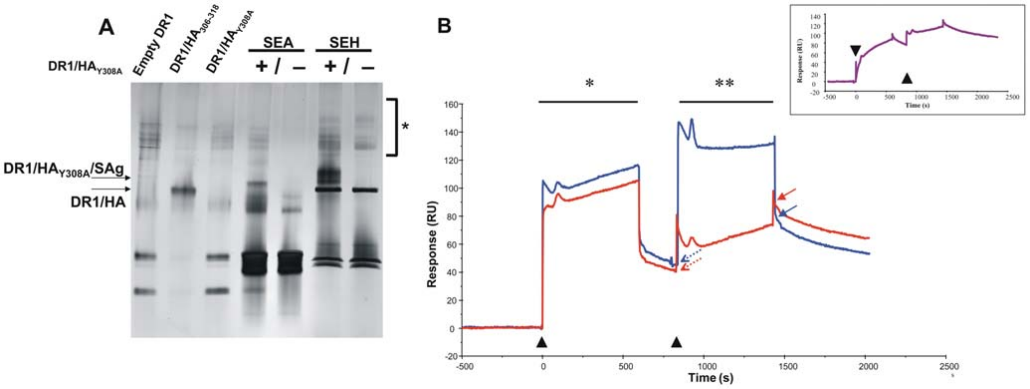
SEA and SEH bind HLA-DR1 in solution. *(A) SEA-DR1 and SEH-DR1 complexes are SDS stable.* Various Enterotoxins in the presence or absence of HLA-DR1/peptide complexes were incubated in PBS with a final SDS concentration of 0.1%, for 5 min at room temperature and subjected to electrophoresis in a 12% polyacrylamide gradient gel. The gel was silver stained by standard protocols. There was no evidence of multimer formation (*) *Lane 1*: empty DR1. *Lane 2*: DR1/HA_306-318_ complex. *Lane 3*: DR1/HA_Y308A_ complex. *Lane 4*: SEA+DR1/HA_Y308A_ *Lane 5*: SEA only *Lane 6*: SEA+DR1/HA_Y308A_ *Lane 7*: SEH only. *(B) SEA-DR1 but not SEH-DR1 complexes can bind an additional DR1*. A solution of DR1 was injected on a HA_306-318_ peptide decorated BIAcore CM5 chip (*) and allowed to dissociate for 3 minutes. A second injection (**) of preformed SEA-DR1 (*red*) resulted in a 30% greater RU change than SEH-DR1 (*blue*), as measured by the difference between the solid and dashed arrows. Two consecutive injections of DR1 alone on a control flow cell (inset) showed the same fold increase in RUs as SEH-DR1. ▴, start of injection.

In order to test the binding and potential crosslinking of recombinant DR1 molecules by SEA in solution, we performed a “gentle SDS” PAGE assay to probe the conformational stability of DR1 complexes [22], [28]. Soluble peptide-DR1 complexes that were SDS-unstable were pre-incubated with SEA or the control Staphylococcal Enterotoxin H (SEH), then incubated with SDS for an additional 2 minutes without boiling, and the solution was run on a gel in the presence of SDS under non-reducing conditions (**Fig. 1A**). SEH is a well-characterized superantigen [2], [29] with a similar overall fold and Zinc dependent binding site as SEA; however, SEH does not possess a second binding site, and is therefore a good “non-clustering” control. The SDS-unstable complexes (lane 3) now attained SDS stability and migrated differently (lanes 4 and 6), suggesting that SEA and SEH bound and conferred rigid SDS stable conformations to otherwise unstable peptide-DR1 complexes. However, there was an absence of supershifted bands (**Fig. 1A**, *) suggesting that no detectable aggregates were formed. In addition, in a size-exclusion experiment where equal amounts of soluble SEA or SEH and soluble DR1 were incubated in the presence of excess ZnCl_2_ or EDTA (to disrupt the Zn^2+^ binding site) and run on a pre-equilibrated size-exclusion column, no high molecular weight peaks corresponding to SEA-DR1 multimers were observed (data not shown).

### Real time binding experiments reveal SEA but not SEH crosslinks DR1

The gel-based experiments could not conclusively demonstrate the crosslinking of soluble recombinant DR1 molecules by SEA. Hence, we attempted to follow the binding of these two molecules in real-time using Surface Plasmon Resonance. In a BIAcore experiment, a solution of DR1 was allowed to bind to HA_306–318_ peptides immobilized on a CM5 chip. After flowing buffer for a short period, a second injection of DR1 pre-incubated with either SEH or SEA at a 1∶1 molar stoichiometry was performed. Given that one of the two binding sites on SEA is a weak binding site of micromolar affinity [1], [21], we hypothesized that there may exist a fraction of SEA in solution that remained bound to DR1 through only one binding site. This would allow SEA-DR1 complexes to bind transiently to the DR1 on the chip via the second binding site on SEA, whereas SEH-DR1 should show little or no such binding. **Fig. 1B** shows the real time raw binding data measured in Response units (RUs) from one of two experiments. There was stable binding of DR1 to the peptide-immobilized surfaces in both flow channels (*). However, in the second binding step (**), SEH-DR1 (blue) showed only a small amount of binding. The RU change was similar to a control flow cell treated with two consecutive DR1 injections (**Fig. 1B**, inset), suggesting that in the SEH-DR1 and control DR1 only group, some binding was seen between free DR1 from the second injection and peptide freshly dissociated from DR1 from the first injection. However, the addition of a molar equivalent of SEA-DR1 (red) to immobilized DR1 resulted in an increase of 55 RUs, which was greater than the RU change observed with SEH-DR1 or DR1 alone (∼40 RUs). Additionally, the slope of the SEA binding curve was greater than the SEH curve, and there was 30% more ligand bound as compared to controls at the end of the injection. The sudden changes in RU at the start of the injection and linear association curves are common SPR artifacts (see Materials and Methods). Thus, at least for a fraction of the molecules present in solution, SEA but not SEH can crosslink two molecules of DR1, forming DR1-SEA-DR1 trimers.

### SEA induces clustering of DR1 on the surface of B cells as measured by FRET

We next attempted to visualize the interaction between SEA and DR1 on the surface of cells. Here, we used Förster Resonance Energy Transfer (FRET) as a tool to visualize the clustering of DR1 on the surface of B cells. The primary advantage of FRET is the ability to dissect molecular interactions that are spatially separated by distances that are much smaller than the resolution offered by light microscopy [30], [31], ∼50 Å for the fluorophore pair used here. The methods and mathematical modeling of the translation of efficiency of FRET into extent of clustering have already been elucidated [30] and are explained briefly in Materials and Methods. In order to monitor SEA-DR1 interactions on cell membranes, we utilized a transgenic mouse that expressed the human MHC class II molecule HLA-DR1 as the only MHC class II molecule on the surface of its antigen presenting cells (DR1 tg mice, [32] and Dalai, S and S. Sadegh-Nasseri, unpublished data). Single cell suspensions of B cells negatively isolated from DR1 tg mice spleens were incubated with or without SAg in the presence or absence of excess EDTA for ∼45 minutes at 37°C. The time of incubation was presumably long enough to allow for membrane clustering events to take place, since in the capping experiments (see below), significant membrane DR1 aggregation had already taken place within 15 minutes (**Supplementary Fig. S1**). The incubation was short enough that internalization of SAg or DR1 itself was minimal [21], [33], [34], [35]. Even at low resolution, it was clear that the addition of SEA did not cause internalization of DR1 on B cells, in agreement with published data from other groups [33], nor did it mediate aggregation of these molecules, whereas the addition of anti-DR1 and polyclonal secondary antibodies at 37°C caused capping of these surface proteins (**Supplementary Fig. S1**).

**Figure 2:**
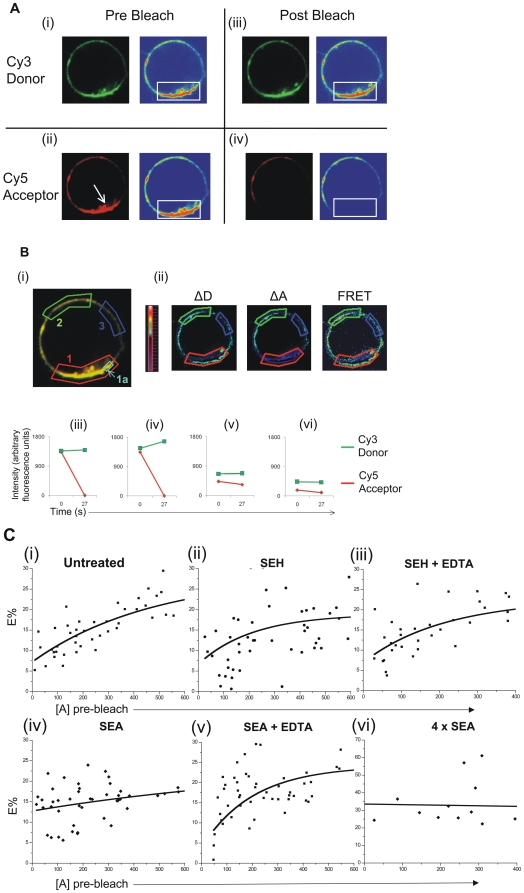
SEA induces clustering of HLA-DR1 on the B cell surface. *(A) Acceptor Photobleach experiments can quantitatively measure energy transfer.* Images of B cells isolated from wtDR1 transgenic mice were stained with Donor (Cy3) and Acceptor (Cy5) conjugated anti-DR monoclonal antibodies (L243) at Donor∶Acceptor ratios of (1∶1) before *(i, ii)* and after *(iii, iv)* acceptor photobleaching, indicated by arrow. Donor channel, *i, iii*; acceptor channel, *ii, iv*. The photobleached area is boxed in the range indicator (to the right of each image). *(B)* FRET in the various regions of interest (ROI) on the cell surface *(overlay, i)* can be quantified from changes in the intensity in the Donor and Acceptor channels *(ii)*. Acceptor photobleach (*red*) results in variable increase in Donor intensity (*green*; ROI 1, *iii*; ROI 1a, *iv*), while control ROIs 2 and 3 (*v,vi*) show little change in donor and acceptor intensities. *(C) SEA induces clustering of HLA-DR1 on B cells*. Energy Transfer (E%) values were plotted against acceptor Fluorescence ([A] pre-bleach) for untreated B cells *(i)*, B cells treated with with SEH +/− EDTA *(ii,iii)*, SEA in the presence of EDTA *(iv)* or B cells treated with 10 µg or 2.5 µg SEA *(v,vi)* for 45 minutes, here, at Donor∶Acceptor molar ratio 1∶4. Data from one of two experiments.

For the cells in each of the groups, FRET assays were performed to gauge the presence of membrane clustering of DR1. Here, DR1 molecules on the B cells were labeled with “donor” fluorophore Cy3-conjugated and “acceptor” fluorophore Cy5-conjugated monoclonal antibodies at various Donor∶Acceptor (D∶A) antibody ratios. We used the exact same monoclonal antibody, L243, against DR1 as donor and acceptor, and added the reagent at well above saturating concentrations to ensure that the FRET measurements would accurately reflect the extent of clustering of that receptor. This is an important point; the bivalency of the whole antibody is rendered irrelevant at saturating doses, so L243 mediated crosslinking of DR1 molecules (which would have been a maximum of two molecules at any rate) is minimal. Also, because we use the same antibody (separately labeled by donor or acceptor fluorophores) against DR1, we neither induce large-scale antibody mediated aggregation of DR1 nor generate false FRET signals, since each DR1 molecule can be bound only by one antibody molecule. An antibody binding experiment showed that there was no significant change in the affinity (Kd) and only a small change in maximal binding (Bmax) of labeled L243 to HLA-DR1 in the presence or absence of enterotoxin (**Table 1**). The addition of SEA over time caused a very modest reduction in the labeling of DR1 by the antibodies (**Supplementary Fig. S2**), suggesting that any changes in receptor density or receptor labeling upon this treatment were minimal. Shown in **Fig. 2** is one cell (in this case, expected to show clustering) out of at least 50 cells that were imaged for each experimental group, in one out of at least two experimental runs. The surface labeling of DR1 was slightly variable across various areas of each cell, but there was no evidence of receptor capping (**Fig. 2A**). In cells imaged before and after photobleaching the acceptor fluorophore (**Fig. 2A**, boxed), some but not all of the photobleached areas showed significant increases in donor intensities, indicative of high DR1 clustering (**Fig. 2B**, iii, iv), while unbleached sections showed no change, as expected (v, vi). The changes in Donor and Acceptor Intensities upon photobleaching were used to compute the FRET efficiency for each region or cell imaged (ii). Plotting the FRET efficiency against the brightness of the acceptor before photobleaching, i.e. the density of acceptor labeled antibody bound to HLA-DR1, for all cells allows for the estimation of the extent of clustering of the HLA-DR1 for each group. Based on the theory detailed in Materials and Methods and [30], an increase in the extent of non-random clustering of HLA-DR1 on the surface of the B cells imaged would result in the following trends in the FRET % vs. [Acceptor] plots: (i) a lack of dependence of FRET % on [Acceptor] irrespective of the D∶A ratio, and (ii) an increase in FRET % with an increase in molar fraction of the donor. For clarity, only the group with D∶A ratio of 1∶4 is shown; the data with D∶A ratios of 1∶1, 1∶2 and 1∶4 are included in **Supplementary Fig. S3**.

**Table 1:** Presence of SEA or SEH does not significantly alter the affinity of the anti HLA-DR1 probe L243.

	K_d_(nM)	B_max_
untreated	7.90±0.41	295.60±3.69
SEH	10.79±0.60	286.95±4.01
SEA	9.70±0.77	260.25±5.15

B cells from DR1tg mice incubated with no enterotoxin or 2.5 µg SEA or SEH for 1 hour at 37°C were stained with various dilutions of Cy3-L243 and analyzed by flow cytometry. Plots of L243 concentration vs. Mean Fluorescence Index were then fitted to a two-step binding equation (see data analysis) to obtain Kd, Bmax values. Standard deviations are in parentheses.

On inspection of the FRET % vs. [Acceptor] graphs for the various groups, some trends are clearly visible. In the absence of SEA treatment (**Fig. 2C**, i) there is an obvious dependence of FRET % on [Acceptor]. The addition of the control SAg SEH does not increase clustering of surface DR1 on B cells in the presence or absence of EDTA (**Fig. 2C**, ii, iii). However, the addition of 10 µg or 2.5 µg SEA per 10^6^ B cells per mL decreases the dependence of FRET % vs. Acceptor density, resulting in lower curvilinearity and a smaller slope in the best fit line that defines the FRET % for a given D∶A ratio (**Fig. 2C**, iv, vi). The efficiency of energy transfer is especially high at the higher dose of SEA, indicating substantial clustering of surface DR1 at this dose of SAg. This increase in clustering is reversed by the inactivation of the Zn^2+^ dependent binding site with EDTA (**Fig. 2C**, v). Inspection of the plots of FRET% vs. various D∶A ratios also support these conclusions. Treatment with SEA causes an increased correlation between FRET % and D∶A ratio, resulting in greater separation between FRET % values, or a greater difference between the intercepts for the fits, for the various ratios. This is reversed by either the addition of EDTA or treatment with SEH (**Supplementary Fig. S3A, B**), suggesting that SEA but not SEH mediates the formation of clusters of DR1 molecules on B cells via both its binding sites.

### SEA does not induce large-scale aggregation of immunogold labeled DR1

**Figure 3:**
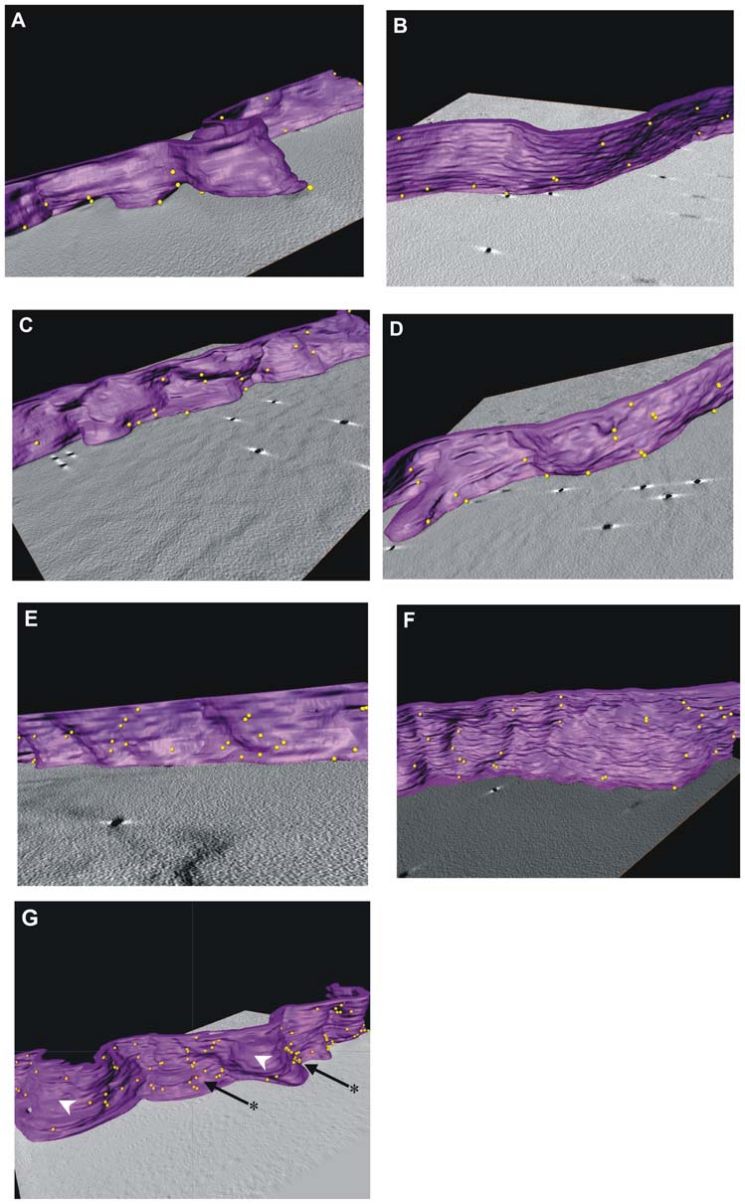
SEA does not mediate large-scale clustering of HLA-DR1 on B cells. B cells treated as in Fig. 2 were stained using anti-HLA-DR primary and gold-labeled secondary antibodies, fixed and analyzed. Representative tomograms of 250 nm thick sections of these cells were generated and segmented to highlight the 3D distribution of DR1 labeled by 6 nm gold beads (*gold spheres)* on the B cell membrane (*purple*). B cells were untreated (*A*), treated with SEA+Zn^2+^ (*B*), SEA+EDTA (*C*) or SEH (*D*). Representative stretches of cell membrane with a higher density of DR1 in cells treated with SEA (*E*), or untreated B cells (*F*) also showed no aggregates. Forced crosslinking of DR1 *(G)* caused aggregation (capping) of DR1 in some areas (black arrow/asterisk) but not others (white arrowhead).

We employed electron tomography and electron microscopy of thick and thin sections respectively, of immunolabeled, fixed, plastic-embedded cells to independently test the conclusions from the FRET experiments, and in addition, gain insights into the size of the clusters induced by SEA. We isolated B cells and treated them with various superantigens in the presence or absence of EDTA as before. We then used excess unlabeled anti-DR1 antibody L243 to bind surface HLA-DR1 on these B cells, followed by fixing and then staining with 6 nm gold bead conjugated secondary antibodies to prevent antibody mediated clustering. To assess the three dimensional surface distribution of DR1 on the surface of B cells, we performed electron tomography on 250 nm thick sections obtained from labeled cells (see Materials and Methods). Electron tomography allows visualization of sub-cellular assemblies in 3D at resolutions higher than light microscopy [25] and is ideally suited to determine the spatial distribution of the 6 nm gold markers at the B cell membrane (**Supplementary Movie S1**). The tomograms generated were segmented to highlight the locations of the gold particles on the surface of treated or untreated cells (**Fig. 3** **, Supplementary Movie S2**). There was no obvious indication of large-scale aggregation or capping of surface DR1 induced by SEA. Indeed, there were no striking differences in clustering between the various control groups (**Fig. 3A–D**). It is noteworthy that even in the stretches of membrane with a high density of gold-labeled DR1 (**Fig. 3E, F**), the addition of SEA did not produce an obvious change in the degree of clustering. In sharp contrast, the addition of primary antibody followed by gold-labeled polyclonal secondary at 37°C caused the formation of large aggregates or capping of MHC class II on the B cell surface (**Fig. 3G**). The capping is not because of unnaturally high surface expression of DR1 because there are areas that have baseline levels of DR1 (arrowheads) immediately adjacent to the areas that have large aggregates of DR1 (arrows with asterisks). This forced capping also caused an increased incidence of membrane ruffling of the cell and internalization of DR1 (**Supplementary Fig. S4A, B**), which was not observed with the SEA treated cells.

**Figure 4:**
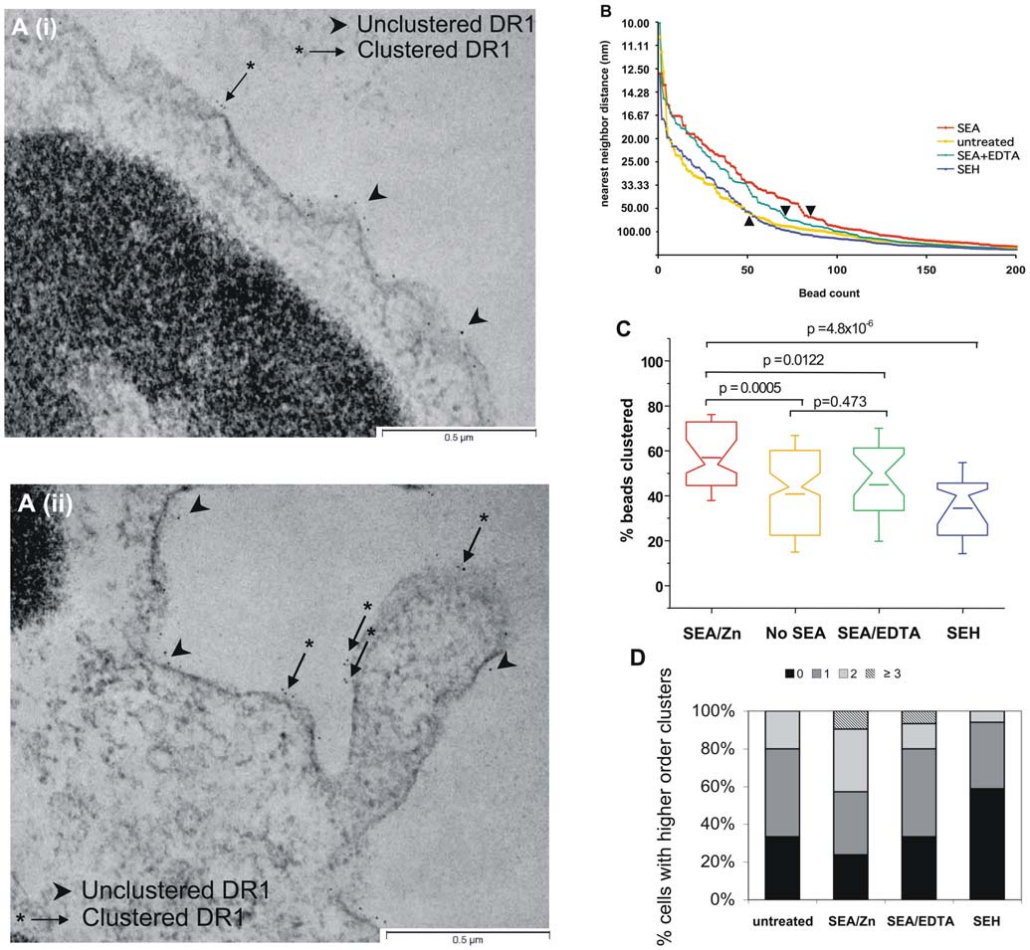
SEA induces small clusters of HLA-DR1 on the B cell surface. B cells were treated, stained and fixed as in Figure 3, and thin sections (80 nm) analyzed by transmission electron microscopy. *(A)* Images of untreated B cells *(i)* or B cells treated with 2.5 µg SEA *(ii)* showed varying degrees of clustering of HLA-DR1. arrow/asterisk, “clustered” gold beads; arrowhead, “unclustered” gold beads. *(B)* Nearest neighbor analysis of B cells treated with SEA (*red*) shows an increased number of closely apposed gold beads compared to B cells treated with no enterotoxin (*yellow*) or SEH (*blue*). SEA+EDTA (*green*) has an intermediate distribution, and these differences disappear when nearest neighbors are separated by large distances. ▴, separation at 75 nm. *(C)* B cells treated with SEA show a statistically significant increase in the fraction of beads that are within 75 nm of each other, when compared to cells that were untreated or treated with SEH or SEA+EDTA. Same color scheme as above. *(D)* SEA treatment does not mediate higher order aggregation of HLA-DR1. Equivalent stretches of B cell membranes of various groups were evaluated for number of instances of “higher order” clusters (clusters of >2 gold beads). There was an increase in the fraction of cells with higher order clusters on SEA treatment, but no dramatic change in the distribution profile between the groups. Data pooled from two experiments.

While there were no dramatic changes in the clustering of gold-labeled DR1 among the various groups of B cells, there were subtle differences in the distribution of gold beads that suggested that inspection of a greater number of cells may give rise to statistically significant differences in clustering between the experimental groups. Since this could be achieved at higher throughput with 2D imaging, we obtained projection electron microscopic images of thin sections (∼80 nm) of the B cells treated as above (**Fig. 4A** i, ii). In a “blinded” experiment, many such slices (>30/group) were examined, and the distances between adjacent gold beads were measured along the contour of the cell membrane. With both the tomographic (3D) and projection (2D) imaging experiments, the addition of SEA or SEH did not appear to have altered the gross morphology or the overall extent of staining of DR1 molecules on the cell membrane (**Fig. 4A**).

A “nearest neighbor” analysis of 200 measurements from each group showed that untreated and SEH treated B cells showed a similar distribution of gold beads with comparable distances between adjacent gold beads (**Fig. 4B** yellow, blue). However, SEA treated cells (red) showed a marked increase of ∼1.8 fold in the proportion of closely apposed gold beads as compared to untreated or SEH-treated cells. The addition of SEA/EDTA (green) showed an intermediate distribution. The differences in gold bead distribution between the various groups were greatest when the distance between nearest neighbors was 60–80 nm, and these differences diminished at greater inter-bead distances. Taking into consideration these data and the approximate molecular sizes of DR1 and SEA, we chose 75 nm as our “clustering cut-off”, i.e., the farthest distance possible between two adjacent gold beads that labeled DR1 molecules actively clustered by SEA (**Fig. 4B**, arrowheads). Thus adjacent beads less than 75 nm apart are counted as “clustered”.

Untreated B cells showed a wide spread in % beads clustered (**Fig. 4C**, yellow). On examination of over 200 cell sections individually, we saw that SEA caused a significant increase in non-random distribution or clustering of HLA-DR1 on the surface of B cells in the presence of Zn^2+^ with a mean of 58% beads clustered (**Fig. 4C**, red). The addition of EDTA to disrupt the zinc-dependent high affinity binding site significantly lowered the proportion of clustered beads, as expected (mean ∼46%), while the addition of the control SAg SEH caused a small, but insignificant decrease in DR1 clustering on the B cells (**Fig. 4C**, green, blue). Finally, we found that the number of higher order clusters on the B cells (clusters with more than two gold beads) was not dramatically altered by the addition of SEA (**Fig. 4D**). There was only a small shift towards higher order clusters; most of the clusters imaged were labeled by 2–4 gold beads, and this was partially reversed by EDTA. We thus conclude that SEA, even in the absence of anti-DR1 antibodies or T Cells, can crosslink DR1 molecules to form small clusters on the surface of B cells.

## Discussion

SEA is an unusual bacterial superantigen in that it has two binding sites for two corresponding sites on the MHC class II molecule. Here, using specific probes in conjunction with powerful imaging techniques of FRET, electron microscopy and tomography, we visualize the generation of small clusters of DR1 molecules on the surface of primary B lymphocytes upon SEA treatment. It is important to clarify here that in using the term “clustering”, we do not imply the covalent cross-linking of various proteins described in this study. Rather, we wish to convey the accretion of SEA and DR1 molecules on the B cell membrane, mediated by moderate to high affinity non-covalent interactions between these two proteins. The term “clustering” has indeed been increasingly utilized to mean as such by Immunologists who study the spatial re-organization of various cell-surface receptors [36], [37]. Our FRET studies showed a high degree of clustering of DR1 molecules induced by SEA; this was dependent on the dose of SEA and the presence of both binding sites. We also observed that FRET efficiency dropped off dramatically at lower concentrations of SEA (data not shown). This sharp increase in MHC class II clustering with increasing SEA concentration was predicted by a cooperative model of SEA-DR binding [13], [38]. Initially, with our electron microscopic studies, there appeared to be only subtle differences in the clustering between the SEA-treated B cells and controls. On the inspection of large areas of the B cell membrane in three dimensions and at nanometer resolution by electron tomography, we observed no large aggregates of gold-labeled DR1 in the SEA treated cells. On the other hand, forced clustering of DR1 by polyclonal antibodies resulted in large aggregates or “capping” of DR1, along with internalization of DR1 into intracellular vesicles and extensive ruffling of the B cell membrane; neither of these were observed with SEA treatment. However, when we inspected large numbers of B cells, there emerged significant differences between the groups. Treatment of B cells with SEA but not SEH resulted in an increase in DR1 clusters, most of which were labeled by no more than 4 gold particles, and this increase in clustering was partially reversed by the inactivation of one of the two SEA binding sites by EDTA.

How can we reconcile the FRET data that suggested strong clustering, with the results from the electron microscopic imaging? We argue that these data are in fact internally consistent; the FRET assay as performed is not sensitive to the size of the DR1 cluster. While we can show that DR1 molecules on the B cell surface are clustered, we cannot establish whether these molecules form a few large clusters, i.e. aggregates of DR1, or many small independent clusters. The electron microscopic imaging experiments suggest that the latter is the case, and strongly argue against extensive aggregation of surface DR1. The precise size of these small clusters cannot be resolved at present because the immunogold experiments only result in the labeling of a small fraction of the total antigen population. A recent study of CD19 clustering on B cells suggested microclusters of anywhere between 100–500 molecules of B Cell Receptors upon activation [39]. However, modeling studies of SEA-DR_2_ trimers [40] reveal an almost orthogonal “kink” in the trimer, such that one can imagine that the extent of cross-linking of DR1 by SEA on a cell membrane could potentially be self-limiting to no more than 4–5 DR1 molecules due to steric constraints. Thus, we conclude that our results can be explained by a model where SEA clusters DR1 to form many small microclusters or “daisy-chain” oligomers [38], but do not result in the coalescence or aggregation of MHC class II molecules on the surface of B cells [12], [21].

How do these observations translate into explaining the functionality of SEA? Firstly, clustering of membrane proteins is an oft-repeated theme in biological signaling, A central event in T cell activation for example, is the formation of the immune synapse (IS), where sustained cell signaling results from an orchestrated set of clustering events of various receptors on both the T cell and Antigen Presenting Cell membranes [43]. It is certainly likely that a similar set of clustering events, in this case on the DR1 expressing B cell membrane, could be initiated by SEA binding and crosslinking DR1 molecules. The intracellular signaling observed in DR1 expressing cells only when they were treated with SEA that had both functional binding sites [19] was construed to mean that SEA could crosslink DR1 molecules, and that this was necessary to initiate cell signaling. Our results are compatible with these hypotheses, and go one step further to show that the SEA-mediated crosslinking results in the formation of small clusters of DR1 on the cell surface. While we required excess SEA to visualize a statistically significant number of instances of these clusters, it must be noted that SEA exerts its toxic effects at far lower concentrations [2], [5], [6]. Thus it is possible that the induction of only a few small-scale clusters per cell by SEA could suffice to initiate downstream intracellular signaling. Given that DR1 molecules are also retained at the cell surface and not endocytosed for long periods of time when bound by functional SEA [35], it is also possible that these clusters, if they continued to exist as stable clusters over time, could also serve to sustain cell signaling. This would explain the downstream signaling seen in cells exposed to very low doses of SEA. Thus, it is certainly tempting to view a single small cluster of 4–5 MHC class II molecules as the smallest “signaling unit” of an Antigen Presenting Cell, although the direct observation of these rare small clusters over time is impossible with current technologies.

In the case of T cells, these cells require only a small number of ligands (here, potentially both SEA and DR1) to trigger T Cell Receptors (TCR) clustering and downstream activation [41], [42]. In this study, we deliberately excluded T cells so as to exclusively study the effect of SEA on B cells expressing DR1 alone. However, we posit that the small-scale clustering of DR1 molecules by SEA that we observed on the surface of Antigen Presenting Cells may serve to increase the local concentration of TCR ligands and thus the avidity of the TCR-SEA-DR1 interaction. The clustering of MHC II molecules to facilitate antigen presentation is not an entirely new concept in Immunology, and indeed has been previously studied in conjunction with lipid rafts [43], [44]. While we have not studied the re-organization of lipids in this study, we subscribe to the possibility that small clusters of TCR-SEA-DR1 may be sufficient to recruit and perhaps cluster signaling molecules, and initiate signaling downstream of TCRs. It is noteworthy that the addition of such a potent T cell activator only results in the formation of small clusters of MHC class II molecules on B cell surfaces. The implicit suggestion from our studies that small-scale membrane receptor clustering might be sufficient to initiate downstream makes it interesting to speculate that in the absence of superantigens, such clustering, possibly even without the formation of large-scale ordered “immune synapses”, could initiate T cell signaling and perhaps explain the sensitivity and non-linearity of the T cell response to low concentrations of antigen. Certainly, in the case of SEA, the clustering of MHC class II molecules on antigen presenting cells by this superantigen may be the underlying mechanism behind the extremely low concentration of SEA required to trigger the hyperactive, and sometimes lethal, immune response.

## Supporting Information

Figure S1 SEA treatment does not cause capping of surface DR1 on B cells. (A) Freshly isolated B cells treated with SEA for various lengths of time and stained for DR1 using L243-FITC showed fairly even receptor distribution around the cell. (B) Cells stained with anti-DR1-FITC and polyclonal secondary antibodies at 37oC showed typical receptor aggregation or “capping”.
(0.56 MB PPT)

Figure S2 SEA treatment does not cause significant internalization of surface DR1 on B cells (A) Cells isolated from the spleens of DR1 transgenic mice and negatively selected on a CD43 column were 90–95% B220+ B lymphocytes. (B) These cells incubated for various time points with 2.5 µg/mL/106 B cells SEA, and stained for surface DR1 with L243-FITC.
(0.10 MB PPT)

Figure S3 SEA but not SEH increases clustering of surface DR1 on B cells. (A) Shown is the data from all the D∶A ratios used for Figure 2c. The addition of SEA causes an increase in dependence of FRET (E%) values to the D∶A ratio, resulting in a greater separation between the groups, which is visually apparent. This separation is markedly reduced in the control groups. (B) The intercepts of the linear fits applied to these data showed a dose dependent increase in E% for SEA treated cells as would be predicted, but not in the control groups. Again, EDTA reduces the SEA induced clustering of DR1.
(0.33 MB DOC)

Movie S1 Acquisition of Tilt series. A thick section of embedded and stained B cell (see Materials and Methods) was imaged at tilt angles ranging from −60° to +60° at 2° increments, cross-correlated and the individual images sequentially output as a movie. The gold fiducials appear as large dark dots, while the 5 nm gold markers which label the surface MHC class II appear as small dots on the cell membrane (the nucleus is on the bottom right). The latter are easier to visualize when the movie is allowed to play at normal speed, and appear most prominently at lower tilt angles, e.g. a group of gold beads near the centre of the image, to the top-left of the small spherical vesicle.
(3.85 MB MPG)

Movie S2 Tomogram. A dataset comprising of images of the thick section of the gold bead decorated B cell taken at different tilt angles was used to generate a tomogram. The same dataset used to make movie S1 was used to generate the volume shown in S2. As one “walks through” the 3D volume, the gold beads appear transiently at high contrast at appropriate z slices. The fiducials appear as the larger black dots towards the start and end of the volume, while the 5 nm gold particles appear throughout the volume and are restricted to the membrane, where they presumably have bound MHC class II molecules
(7.26 MB MPG)

Movie S3 Image segmentation. The tomogram generated above was segmented by Amira to highlight a section of the B cell membrane (purple) and the 3D distribution of some of the gold bead labeled DR1 molecules. Gold spheres with a diameter of ∼15 nm were placed at the xyz coordinates corresponding to location of the gold beads in the tomogram. For clarity, the other aspects of the tomogram such as the nuclear membrane etc were left unhighlighted. Still images from segmented 3D volumes such as these are represented in Figure 3 in the main manuscript.
(5.81 MB MPG)
