# Supplementary material for: Staphylococcal Enterotoxin A Induces Small Clusters of HLA-DR1 on B Cells
Source: PLoS One. 2009 Jul 9;4(7):e6188. doi: 10.1371/journal.pone.0006188 (PMC2705189; doi:10.1371/journal.pone.0006188)
Supplement: Supplementary file 1 [file pone.0006188.s003.doc]

## Slide 1

Supporting Figure S3A

untreated

SEH

SEH + EDTA

(i)

(ii)

(iii)

(iv)

(v)

(vi)

SEA + EDTA

SEA

4 x SEA

1:1

1:2

1:4

D:A ratio

[A] pre-bleach

[A] pre-bleach

## Slide 2

Supporting Figure S3B

average increase in intercept (E%)
